# The association between prenatal exposure and childhood asthma: the mediating role of gut microbiota

**DOI:** 10.3389/fmicb.2025.1664708

**Published:** 2025-09-17

**Authors:** Zidi Ning, Ye Zhang, Ruoyu Lu, Anda Zhao, Zeyu Wang, Jiajun Yuan, Shenghui Li

**Affiliations:** ^1^School of Public Health, Shanghai Jiao Tong University, Shanghai, China; ^2^School of Computer Science, Faculty of Engineering, The University of Sydney, Sydney, NSW, Australia; ^3^Hainan Branch, Shanghai Children’s Medical Center, School of Medicine, Shanghai Jiao Tong University, Sanya, China; ^4^Shanghai Engineering Research Center of Intelligence Pediatrics (SERCIP), Shanghai Children’s Medical Center, affiliated with School of Medicine, Shanghai Jiao Tong University, Shanghai, China; ^5^Child Health Advocacy Institute, National Children’s Medical Center, Shanghai Children’s Medical Center, affiliated with School of Medicine, Shanghai Jiao Tong University, Shanghai, China; ^6^MOE-Shanghai Key Laboratory of Children’s Environmental Health, Shanghai Jiao Tong University School of Medicine, Shanghai, China

**Keywords:** childhood asthma, gut microbiota, prenatal exposure, maternal exposure during pregnancy, wheeze

## Abstract

**Background:**

Asthma is a common chronic respiratory disease that usually originates in early childhood. Emerging evidence implicates the gut microbiota as a modulator in asthma development, with growing attention to the interplay between prenatal exposures, maternal/offspring gut microbiota, and subsequent asthma risk. However, no comprehensive review has systematically examined the relationships.

**Objective:**

This review aimed to explore whether the gut microbiota acts as a mediating factor in the association between prenatal exposure and childhood asthma.

**Results:**

A systematic search was performed in the PubMed, Scopus, and Web of Science databases up to March 1, 2025, employing keywords related to childhood asthma, gut microbiota, and prenatal exposure. Only population-based studies were considered. Eight studies met the inclusion criteria. Among which, two focused on pet exposure during pregnancy, three on delivery mode, two on the combined effects of delivery mode and antibiotic exposure, and one on maternal diet. Exposure to pets during pregnancy may result in distinct microbiota profiles in the offspring, which may potentially confer a protective effect against asthma. Antibiotic use and cesarean delivery were associated with increased asthma risk. Conversely, high maternal fecal short-chain fatty acid levels appeared protective against childhood asthma development. The gut microbiota may play a mediating role in these associations.

**Conclusion:**

Prenatal factors significantly correlate with offspring gut microbiota and early immune development, thereby affecting asthma susceptibility. Further studies are needed to expand prenatal exposure assessments and elucidate the specific mechanisms by which the gut microbiota mediates the association between prenatal exposures and childhood asthma.

## Graphical abstract

Pharmacological mechanisms of GE and its active ingredients.
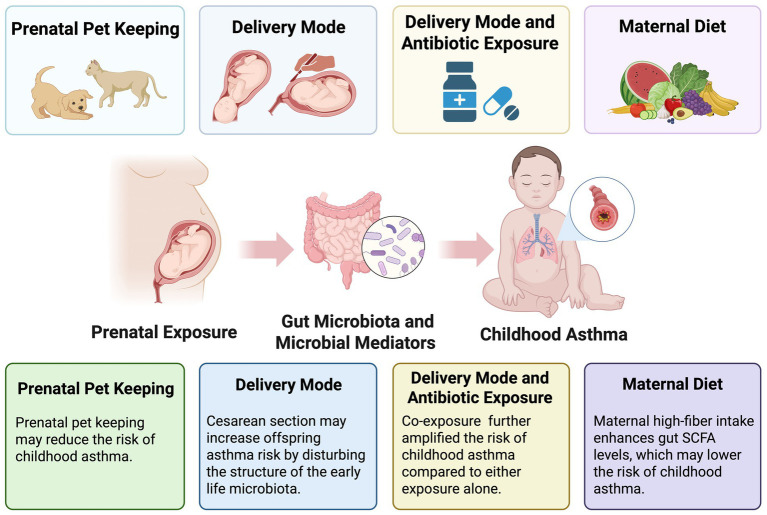

## Introduction

Asthma is a chronic respiratory condition characterized by recurrent wheezing, shortness of breath, and chest tightness (Sockrider and Fussner, 2020). Epidemiological data showed that its prevalence ranged from 9.1 to 13.7% worldwide (Asher et al., 2020; García-Marcos et al., 2022), with the majority of countries continuing to demonstrate an upward trend (Stern et al., 2020). Asthma typically initiates during childhood and is triggered by allergens (Miller et al., 2021). T helper 2 cells (Th2 cells) responses play a central role in asthma pathogenesis, by activating cytokines like interleukin (IL)-4, IL-5, IL-9, and IL-13, which promote eosinophil infiltration into airway wall and excessive mucus production (Hammad and Lambrecht, 2021).

In recent years, evidence indicates that the abundance and diversity of gut microbiota correlate with childhood asthma susceptibility (Arrieta et al., 2015; Sordillo et al., 2019). Children with asthma exhibited decreased levels of beneficial bacteria such as *Lactobacilli* and *Clostridium*, alongside increased colonization by potential pathogens such as *Bacteroides fragilis* and *Candida albicans* (Kahhaleh et al., 2024; Budden et al., 2017; Chiu et al., 2023). Moreover, reduced abundance of microbiome-encoded carbohydrate-active enzyme genes was observed in the gut of asthma children, which may diminish butyrate production and negatively correlate with mite-specific IgE responses (Chiu et al., 2023). All these findings underscore the immunomodulatory potential of gut microbiota during childhood (Kahhaleh et al., 2024). Moreover, emerging findings have revealed that environmental exposures during pregnancy alter both the maternal gut microbiome and the establishment of the offspring’s gut microbiota (Xiao and Zhao, 2023). Robust association has been established that microbial alterations induced by prenatal exposures contribute to individuals’ immune-inflammatory responses and asthma pathogenesis (Budden et al., 2017). A 2021 review has synthesized evidence linking prenatal gut microbiota to offspring’s allergic diseases, suggesting that maternal gut microbiota plays a fundamental role in priming the developing immune system to acquire immune competence (Gao et al., 2021).

Of note is that no reviews have summarized whether prenatal environmental factors mediated maternal and/or offspring microbiome, thereby influencing childhood allergic outcomes. Given the context-dependent nature of gut microbiome alterations and the disease-specific effects of immune responses, there is a critical need to clarify the influence of prenatal exposures on microbiota composition and their subsequent effects on discrete allergic outcomes, such as asthma.

The purpose of this review is to investigate whether microbes act as an intermediate factor linking prenatal exposure to offspring asthma development, with particular focus on exposure-mediated alterations in maternal and offspring gut microbiota.

## Methods

### Data source and search strategy

This scoping review was conducted following the Preferred Reporting Items for Systematic Reviews and Meta-Analyses Extension for Scoping Reviews (PRISMA-ScR) guidelines. We searched the PubMed, Scopus, and Web of Science databases up to March 1, 2025 using a combination of terms related to “asthma or respiratory conditions or wheezing,” “gut microbiota or intestinal flora,” “pregnancy or maternal exposure,” and “epidemiological or population-based studies.” No restrictions were applied regarding publication date or language. Reference lists of eligible studies were also reviewed for additional relevant studies.

### Data management and study selection

All records were imported into EndNote (Clarivate Analytics). After removing duplicates, study selection was conducted in three stages: title screening, abstract review, and full-text evaluation. Two independent reviewers screened each study based on predefined eligibility criteria, with discrepancies resolved through discussion or by a third reviewer.

### Inclusion and exclusion criteria

Studies were included if they met the following criteria: (1) Population-based studies with recruitment of both mothers and children. (2) Exposure assessment was restricted to the period spanning pregnancy through delivery. (3) Stool sample collection and microbial analysis from mothers and/or infants. (4) Documented childhood asthma outcomes or wheezing. (5) Included analysis of mediating role of the gut microbiota.

A total of 1,527 records were initially retrieved from PubMed (*n* = 147), Scopus (*n* = 548), and Web of Science (*n* = 832). After removing 414 duplicates, 1,113 records remained for screening. Following title and abstract screening, 789 were excluded. Subsequently, 324 full-text articles were sought for retrieval and further assessed. Of these, 316 were excluded due to not meeting the eligibility criteria upon full-text review. Ultimately, 8 articles met the inclusion criteria and were included in the present review. The study selection process is illustrated in the PRISMA flow diagram.

### Data extraction

Two researchers extracted all the data independently. Each reviewer extracted the following from each study: name of the first authors, publication year, country of study, study aims, study design, study subjects, exposure factors, assessment of child outcomes, covariates and principal results. Disagreements were settled through consensus.

## Subsections relevant for the subject

### Description of studies

**Figure 1:**
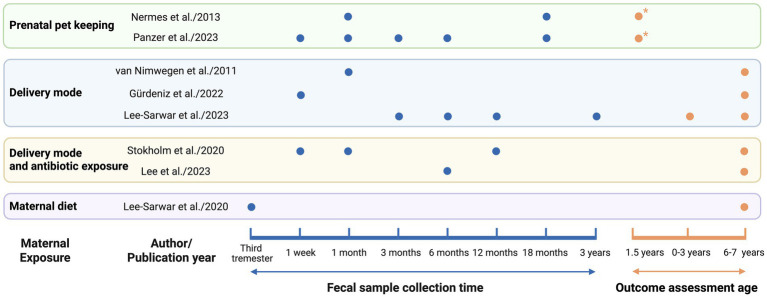
Timepoints of fecal sampling and asthma outcome assessment. Blue dots (●) indicate fecal sample collection for gut microbiota analysis. Orange dots (●) represent timepoints of asthma outcome assessment. Orange dots with an asterisk (●*) indicates wheeze assessment, which is commonly regarded as an early clinical manifestation of asthma.

Table 1 presents the detailed data extracted from all included epidemiological studies (*N* = 8). Based on exposure during pregnancy, the studies were classified into four categories: pet exposure (*n* = 2), mode of delivery (*n* = 3), the combined effects of delivery mode and antibiotic exposure (*n* = 2), and maternal diet during pregnancy (*n* = 1). All included studies employed a cohort design. Geographically, these studies encompassed diverse populations across three continents. Four were conducted in Europe, specifically, one each in Finland and the Netherlands, and two in Denmark. Three studies originated from North America (United States), and one was conducted in Asia (South Korea). In terms of publication timelines, six were published within the past 5 years, while the remaining two were published in 2011 and 2013. Regarding fecal sample collection, four studies performed a single collection, whereas the other four utilized multiple time points. For outcome assessment, two evaluated wheeze, a common early clinical manifestation of asthma; one assessed early asthma at the age of 0–3 years and asthma at 6–7 years; and the remaining five focused exclusively on asthma aged 6–7 years. The information is depicted on sample collection timing and outcome measurement periods from eight studies (Figure 1), along with a summary of adjusted covariates (Table 2).

**Table 1:** Summary of epidemiological studies on prenatal exposures, gut microbiota, and childhood asthma.

Author, year, and country	Design	Participants	Aims	Exposure	Outcome	Covariates	Principal results
Prenatal pet keeping							
Nermes et al. (2013), Finland	Birth cohort	Total: 129 maternal–child pairs	Investigating the interrelationship between perinatal pet exposure, doctor’s diagnosed wheezy bronchitis (WB), and compositional changes in the gut microbiota of infants	Perinatal exposure to indoor pets (mainly cats and dogs) Pet exposure: *N* = 30 Pet-free: *N* = 99	Evaluation time: Within 24 months Outcomes: None of the 30 infants perinatally exposed to pets had suffered from wheezy bronchitis by 24 months, while 15(15%) of the 99 non-exposed infants had	Maternal asthma, maternal atopy, parental smoking, duration of breast feeding, study group, number of siblings, gender	None of the 30 pet-exposed infants developed wheezy bronchitis (WB), compared to 15% of non-exposed infants (*p* = 0.03). *B. longum* was more abundant in non-wheezing, pet-exposed infants, while *B. breve* was higher in wheezing, non-exposed infants (both *p* = 0.02)
Panzer et al. (2023), United States	Birth cohort	Total: 141 mother–child pairs	Determining whether infants born into homes with indoor dog(s) exhibit altered gut microbiome development	Prenatal dog-keeping: *N* = 75 Pet-free: *N* = 56	Evaluation time: 18 months of age Outcomes: Seven had parentally-reported, doctor diagnosed asthma (five dog vs. two pet-free)	Maternal race, household income, maternal age at birth, mode of delivery, child sex and first-born child, and includes a term for the dog × time interaction	Dog-exposed infants showed higher gut microbial phylogenetic diversity up to 18 months, with significant differences at 3 and 6 months (*p* = 0.041). After covariate adjustment, *Fusobacterium* was significantly enriched in infants exposed to dogs during pregnancy
Delivery mode							
van Nimwegen et al. (2011), The Netherlands	Birth cohort	Total: mother: *n* = 2,834 Children: *n* = 2,733	Investigating the relationship between microbiota composition, mode and place of delivery, and atopic manifestations	Vaginal delivery at home: *N* = 1,179 (46.1%) Vaginal delivery in the hospital: *N* = 1,092 (42.7%) Cesarean section delivery in the hospital: *N* = 288 (11.2%)	Evaluation time: Asthma at the age of 6 to 7 years Outcomes: Vaginal delivery at home: *N* = 44/854 Vaginal delivery in the hospital: *N* = 67/792 Cesarean section delivery in the hospital: 17/216	Maternal education, duration of breastfeeding, maternal smoking during pregnancy, presence of pets, number of siblings, gender, and season of birth	Cesarean-delivered infants had reduced *Bifidobacterium* and *Bacteroides*, but increased *Clostridium difficile* due to lack of maternal microbial exposure. *Clostridium difficile* was linked to higher asthma risk at age 6–7 and partially mediated delivery-related asthma/allergy effects
Gürdeniz et al. (2022), Denmark	Birth cohort	Total: COPSAC2010: 738 pregnant women and their 700 children COPSAC2000: A mother–child cohort of 411 children born to mothers with asthma history	Elucidating the link between birth by cesarean section and asthma using newborn metabolomic profiles and integrating early-life gut microbiome data and cord blood immunology	Natural birth: *N* = 533 (79%); *N* = 309 (80%) Emergency cesarean section: *N* = 79 (12%); *N* = 49 (13%) Elective cesarean section: *N* = 64 (9%); *N* = 29 (7%)	Evaluation time: Early asthma at 0–3 years, Asthma at 6 years Outcomes: Unavailable	Sex, gestational age, season of birth, breastfeeding, intrapartum antibiotics	Cesarean-delivered newborns exhibited reduced levels of indoleacetic acid and both primary and secondary bile acids, which were associated with lower abundances of *Bifidobacterium* and *Bacteroides*. These metabolic profiles correlated with higher asthma risk (HR = 1.08–1.21) and were significantly associated with reduced cord blood Treg levels (*R* = 0.37, *p* = 0.003)
Lee-Sarwar et al. (2023), United States	Birth cohort	Total: 657 mother–child pairs	Identifying associations between features of the prenatal and early life fecal microbiomes and child asthma phenotypes	Unavailable	Evaluation time: from birth to age 6 years Outcomes: active asthma at age 6 years: *N* = 115.19%, transient asthma: *N* = 90.15% Early asthma (onset before age 3 years): *N* = 185.28% No asthma: *N* = 405.66%	Breastfeeding, antibiotics, dog ownership, study center, race/et hnicity	Infants born by cesarean section exhibit a pronounced reduction in fecal *Bacteroides* at age 3–6 months (Wilcoxon rank sum test, *p* < 0.001), which impairs the synthesis of microbial sphingolipids such as 3-ketosphingosine (Spearman rho = 0.63 and 0.68, *p* < 0.0001). This metabolic pattern was associated with early asthma risk and a high-risk microbiome trajectory
Delivery mode and antibiotic exposure							
Stokholm et al. (2020), Denmark	Birth cohort	Total: 700 mother–child pairs	Analyzing the effects of delivery mode on the colonization patterns of the gut during the first year of life and to explore whether perturbations of the gut microbiota could explain the delivery mode–associated risk of developing asthma during childhood	Cesarean section:22%, *N* = 151 Vaginal delivery:78%, *N* = 549	Evaluation time: Asthma—like episodes at 0–3 years, asthma at 6 years Outcomes: Total *n* = 48, cesarean section: 13%, *N* = 18, vaginal deliver: 6%, *N* = 30	Gestational age, maternal asthma status, intrapartum antibiotic treatment, induction of birth, maternal pre-pregnancy BMI, antibiotic treatment of the child in the first year of life, number of antibiotic treatments in the first year, older children in the home, pet ownership	Cesarean-delivered infants show reduced *Bacteroides* and *Bifidobacterium*, and increased *Enterococcus* and *Veillonella*. at 1 year, persistence of this cesarean-associated microbial signature was linked to higher asthma risk by age 6 (HR = 2.11, *p* = 0.022). Lower microbiota maturity and reduced cytokine responses (e.g., IL-4, IL-13) further suggest impaired immune regulation mediating this association
Lee et al. (2023), Korea	Birth cohort	Total: 789 children	Identifing the individual and combined effects of prenatal antibiotic exposure and delivery mode on the development of asthma in children and the potential mechanisms underlying these associations	Prenatal antibiotic exposure (−) and Spontaneous delivery: *N* = 447 Prenatal antibiotic exposure (−) and Cesarean section: *N* = 237 Prenatal antibiotic exposure (+) and Spontaneous delivery: *N* = 51 Prenatal antibiotic exposure (+) and Cesarean section: *N* = 28	Evaluation time: Asthma at age 7 years Outcomes: Prenatal antibiotic exposure (−) and spontaneous delivery: *N* = 9/447 Prenatal antibiotic exposure (−) and Cesarean section: *N* = 6/237 Prenatal antibiotic (+) and spontaneous delivery: *N* = 1/51 Prenatal antibiotic (+) and Cesarean section: *N* = 3/28	Family history of any allergic diseases, sex, maternal education level, maternal age at delivery, feeding patterns in the first 6 months, exposure to smoking	Prenatal antibiotic (aOR = 5.70, 95% CI: 1.25–22.81) and cesarean section (aOR = 1.57, 95% CI: 1.36–6.14) were independently associated with childhood asthma, with a synergistic effect when combined (aOR = 7.35, 95% CI: 3.46–39.61; interaction *p* = 0.03). Infants exposed to both had higher Clostridium abundance, while those not exposed to antibiotics had more Enterococcus and Lactobacillus. Cesarean delivery increased Escherichia and Kosakonia levels. Combined exposure was also linked to small-airway dysfunction (*p* = 0.007), suggesting early airway remodeling
Maternal diet							
Lee-Sarwar et al. (2020), United States	Birth cohort (derived from VDAART RCT)	Total: 150 mother–child pairs	Investigating the association between short—chain fatty acids (SCFA) during pregnancy and the risk of asthma and/or allergy in offspring	Maternal fecal short-chain fatty acid levels in late pregnancy and diet	Evaluation time: Asthma at age 6 years old Outcomes: Unavailable	Early pregnancy BMI, breastfeeding	A 10% increase in maternal fecal acetic acid was linked to a 54% lower risk of allergic asthma/wheezing in offspring by age 6 (OR = 0.46, 95% CI 0.22–0.94, *p* = 0.036). No associations were found between absolute SCFA levels or other SCFAs (butyric and propionic acids) and allergic outcomes. Maternal fiber intake correlated positively with total SCFAs, acetic acid, and propionic acid (*p* < 0.05), but not directly with offspring allergy risk. Gut microbiota composition (e.g., Bacillus and Streptococcus) correlated with SCFA levels and inversely with offspring allergic asthma risk

BMI, Body mass index.

**Table 2:** Covariates considered in studies on prenatal exposure and childhood asthma.

Frequency	Covariates*
5	Breastfeeding, gender
3	Presence of pets
2	Maternal asthma status, parental smoking, study center, number of siblings, maternal age at birth, race/et hnicity, season of birth, maternal education level, intrapartum antibiotic treatment, gestational age
1	Maternal atopy, mode of delivery, household income, first-born child, antibiotics, induction of birth, maternal pre-pregnancy BMI, early pregnancy BMI, antibiotic treatment of the child in the first year of life, number of antibiotic treatments in the first year, older children in the home, exposure to smoking, feeding patterns in the first 6 months, family history of any allergic diseases

*Similar covariates from different studies are combined to improve the clarity of the table. BMI, Body mass index.

### Association of prenatal exposures, maternal/offspring gut microbiota, and childhood asthma risk

#### Prenatal pet keeping

Nermes et al. conducted a nested observational cohort study within a randomized controlled trial. In this study, 129 mothers and infants with indoor pet exposure data were analyzed to determine whether perinatal exposure to indoor pets influenced infant gut microbiota composition and the risk of developing wheezy bronchitis by 24 months of age (Nermes et al., 2013). Among 129 infants, 30 had been exposed to indoor pets (mainly cats and dogs), while 99 had not. None of the pet-exposed infants developed wheezy bronchitis, whereas 15 of the non-exposed infants did. Fecal samples collected at 1 month showed that *Bifidobacterium longum* was significantly enriched in non-wheezing, pet-exposed infants, while *Bifidobacterium breve* was more abundant in wheezing, non-exposed infants.

Panzer et al. conducted a longitudinal analysis observing the impact of living with indoor dogs during pregnancy on infant gut microbiota and the risk of allergic diseases including asthma at 18 months of age (Panzer et al., 2023). Among the participants, 81 mothers had been exposed to indoor dogs at least 12 h/day for over 6 months before pregnancy and throughout the whole pregnancy, and another 60 had no pets at home. Of their offspring, four and three were allergic to common inhalants, five and two were diagnosed with asthma, 24 and 11 reported eczema/atopic dermatitis, in the dog-exposed and non-exposed groups, respectively. *Fusobacterium* was found to be pronouncedly enriched in children whose mother underwent dog exposure during pregnancy.

The findings of the two studies support the broader argument that prenatal pet exposure may shape infant gut microbiota, potentially conferring protection against early-life wheezing and influencing long-term susceptibility to atopic, allergic, and asthmatic conditions.

#### Delivery mode

Three studies specifically focused on delivery mode. Van Nimwegen et al. used data from the Dutch KOALA birth cohort to investigate how delivery mode and setting influence infant gut microbiota composition and the subsequent asthma/allergy risk (van Nimwegen et al., 2011). The study included 2,733 children, of whom stool samples were available for 952 infants at 1 month, and asthma was assessed at ages 6–7. It was found that cesarean delivery reduced the colonization of beneficial genera including *Bifidobacterium* and *Bacteroides* and increased colonization by *Clostridium difficile*. Crucially, *Clostridium difficile* colonization was significantly associated with higher risks of asthma, eczema, and food allergy at age 6–7 years. *C. difficile* colonization partially mediated the effects of delivery mode/setting on allergic outcomes. Vaginal home delivery was associated with lower risks of asthma and food sensitization compared to vaginal hospital delivery, particularly in children with an atopic family history.

Gürdeniz et al. analyzed data from two cohorts of the Copenhagen Prospective Studies on Asthma in Childhood (COPSAC) established in 2010 and 2000, including 738 pregnant women and their 700 children from the COPSAC2010 cohort, as well as 411 full-term infants born to mothers with asthma from the COPSAC2000 cohort. Cord blood samples were collected from umbilical vein, dried blood spot (DBS) samples were, respectively, collected at age 2–3 days and 1–12 days in two cohorts, and fecal samples were both collected in infants 1 week after birth. The diagnoses of early asthma and asthma were made at ages 0–3 years and 6 years (Gürdeniz et al., 2022). DBS analysis revealed distinct metabolic profiles between cesarean-born and vaginally delivered infants that indoleacetic acid, an intermediate of tryptophan metabolism, as well as primary and secondary bile acids were lower in cesarean section newborns. The differences in DBS metabolic profile were correlated with varied abundance of *Bifidobacterium* and *Bacteroides* in infant gut. Moreover, the metabolic profiles of cesarean-born infants were positively associated with asthma risk up to ages 6. The frequency of cord blood regulatory T cells (Tregs) was also significantly linked to cesarean-associated metabolic profiles. Summing up all these results, it was suggested that cesarean section can lead to early microbiota-associated metabolic disruptions, thereby increasing asthma risk.

Using data from the Vitamin D Antenatal Asthma Reduction Trial (VDAART), Lee-Sarwar et al. investigated the association of mode of delivery, fecal microbiome with childhood asthma phenotypes among 657 maternal–infant pairs (Lee-Sarwar et al., 2023). Fecal samples were collected at 3–6 months, 1 year, and 3 years. Childhood asthma was classified into three phenotypes: early asthma or recurrent wheeze by age 3, transient asthma (early asthma without symptoms at age 6), and active asthma at age 6. The fecal abundance of *Bacteroides* was observed to be decreased significantly in infants born by cesarean section at 3–6 months of age, which was further linked to higher risk of early asthma and transient asthma. Meanwhile, the reduction in *Bacteroides* was also linked to decreased levels of microbial sphingolipids at 3–6 months of age and then associated with a higher risk of early asthma. Additionally, fecal linoleic acid levels at 1 year were lower in children who developed active asthma by age 6. Although an association was not established between fecal metabolites at age 3 and asthma, the overall data support a potential pathway by which delivery mode influences childhood asthma susceptibility via alterations in the gut microbiome.

#### Delivery mode and antibiotic exposure

Two studies explored the combined effects of antibiotic exposure and delivery mode. Stokholm et al. used data from COPSAC 2010 cohort to investigate whether gut microbial perturbations associated with delivery mode were linked to asthma risk within the first 6 years of life (Stokholm et al., 2020). Fecal samples were collected at 1 week, 1 month, and 1 year of age. At 1 week and 1 month, cesarean-born infants exhibited lower abundance of *Bacteroidetes* and *Actinobacteria* and higher *Firmicutes* and *Proteobacteria* at the phylum level. By 1 year, cesarean-born infants still had higher *Enterobacteriaceae* and *Escherichia/Shigella* at the genus level. Moreover, children born via cesarean section whose intestinal microbiota retains the cesarean characteristics at the age of 1 year experienced significantly more asthma-like episodes at ages 2–3 compared to vaginally delivered children and cesarean section-born children with low cesarean microbiome scores. These children also show a lower overall immune mediator response during acute airway symptom onset and faced an increased risk of asthma by age 6. This study also considered the potential effect of antibiotic exposure since all sampled cesarean section mother received intrapartum antibiotics at delivery, while only 13% for vaginally birth. Although antibiotic use can shift the microbiome structure toward that of cesarean-delivered children, delivery mode remains the primary factor influencing gut microbes and higher childhood asthma risk.

Lee et al. conducted a prospective study based on 789 children from the COCOA birth cohort (Lee et al., 2023). Fecal samples were collected from 207 6-month-old infants, and asthma was diagnosed at age 7. Both prenatal antibiotic exposure and cesarean section were independently associated with an increased risk of asthma. Co-exposure showed an even greater effect, with a dose–response relationship found between the higher antibiotic exposure and childhood asthma in cesarean-born children. Moreover, children born by cesarean section with prenatal antibiotic exposure manifested greater airway dysfunction compared to those with spontaneous delivery without prenatal antibiotic exposure. In addition, an increased relative abundance of *Enterococcus* and *Lactobacillus* was observed in infants without prenatal antibiotics, regardless of delivery mode. Meanwhile, the abundance of *Escherichia* and *Kosakonia* was significantly higher in cesarean section born infants compared to those delivered vaginally, and cesarean-born infants with prenatal antibiotics exposure had the higher level of the relative abundance of *Clostridium*. Collectively, these findings suggest that prenatal antibiotic exposure and cesarean section may lead to childhood asthma development, potentially through changes in early-life gut microbiota.

#### Maternal diet

Lee-Sarwar et al. conducted a secondary cohort analysis within the VDAART trial to examine the association between maternal fecal short-chain fatty acids (SCFAs) in late pregnancy and offspring risk of asthma and allergic outcomes by age 6 (Lee-Sarwar et al., 2020). Among 150 mother–child pairs, the study found that higher relative concentrations of acetate cetic acid in maternal stool were significantly associated with a reduced risk of combined asthma/wheeze and allergic sensitization in children. In contrast, absolute SCFAs levels showed no significant association with individual allergic outcomes. Maternal dietary fiber intake was positively correlated with total SCFAs levels, particularly acetic and propionic acids, suggesting that microbial fermentation of fiber may underlie this association. Furthermore, maternal gut microbiota profiling revealed that specific taxa such as *Eubacterium dolichum* and *Streptococcus anginosus* were linked to SCFAs composition and directionally associated with asthma risk. These findings highlight the potential role of maternal diet–microbiota–metabolite interactions during pregnancy in shaping immune-related disease susceptibility in offspring.

## Discussion

### Prenatal pet keeping

Previous epidemiological studies have indicated a potential relationship between maternal pet exposure and the risk of childhood allergic diseases, such as eczema (Eapen et al., 2022), food allergy (Smejda et al., 2020), and asthma (Lodge et al., 2012). Meanwhile, it has been found that pet exposure during the preconception period can influence the human microbiome, thereby exerting a significant influence in programming offspring’s immune system development (Aichbhaumik et al., 2008).

Nermes et al. performed the first study demonstrating that prenatal pet exposure in urban infants was associated with a decreased risk of asthmatic bronchitis and concurrent alterations in gut microbiota composition (Nermes et al., 2013). The study identified two distinct bacterial patterns: *Bifidobacterium longum* was more abundant in non-asthmatic, pet-exposed infants, whereas *Bifidobacterium breve* was enriched in asthmatic infants without pet contact. Notably, a potentially important dichotomy in *Bifidobacterial* effects was identified. Although *Bifidobacteria* are generally recognized for their anti-inflammatory and immunoregulatory roles (Cukrowska et al., 2020), the study suggests that different species within this genus may exert opposing influences on asthma development.

Another study included in this review showed that prenatal pet exposure correlated with a higher diversity of infant intestinal microbiota, particularly enriched abundances of *Collinsella stercoris, Ruminococcus, Lachnospiraceae*, and *Clostridiaceae* (Panzer et al., 2023). Similarly, *Ruminococcus* levels were found to be elevated in 3–4-month-old infants exposed to furry pets (Tun et al., 2017). *Ruminococcus* and *Clostridium* are key gut microbes involved in fermenting complex carbohydrates to produce SCFAs, such as butyrate and propionate (Li et al., 2025; Adjele et al., 2024). These SCFAs support health by strengthening the intestinal barrier and serving as an energy source for epithelial cells (Rowland et al., 2025). Such microbiome–host interactions may regulate immune development and lower the risk of diseases like asthma (Alswat, 2024). However, due to the limited sample size and the relatively short follow-up period of less than 2 years, it remains challenging to determine whether the observed microbial changes associated with pet exposure are implicated in infant allergy outcomes. In addition, the mixed exposure of multiple pets may introduce confounding effects, further complicating the interpretation of results.

Notably, prenatal and postnatal pet exposures often co-occur. Tun et al. found that their co-exposure was associated with more profound microbial alterations compared to prenatal-only exposure (Tun et al., 2017). Specifically, prenatal-only exposure was associated with transient alterations in specific microbial taxa and abundance ratios, whereas sustained exposure was linked to broader and more stable modifications of the gut microbiota, including enrichment of key commensal genera such as *Ruminococcus* and *Oscillospira*, as well as the suppression of potentially pathogenic taxa. These findings underscore the necessity of accounting for persistent exposure throuthout prenatal and postnatal period in future research.

With pet ownership on the rise (Kretzler et al., 2022), further research is needed to clarify how different types and timings of pet exposure during pregnancy involve in infant microbiome and influence childhood asthma and allergy risks, and to disentangle the respective and combined effects of prenatal and postnatal exposures through longitudinal designs.

### Delivery mode

Accumulating studies found that the mode of delivery influences the establishment of the neonatal gut microbiome (Dominguez-Bello et al., 2010). Notably, delivery mode not only alters microbial diversity but also modulates metabolite profiles, both of which exert a crucial role in early immune development (Sarkar et al., 2021). Vaginally delivered infants exhibit higher levels of Gram-negative bacteria and lipopolysaccharide (LPS), a bacterial component that activates immune response. Conversely, cesarean-born infants lack this LPS-mediated immune activation, which may potentially increasing lifelong susceptibility to chronic diseases (Wampach et al., 2018).

Studies included in this review revealed that cesarean delivered infants exhibited reduced levels of *Bacteroides* and its metabolite sphingosine (Lee-Sarwar et al., 2023). They also showed decreased *Bifidobacteria*, leading to reduced production of tryptophan metabolites and bile acids (Gürdeniz et al., 2022). And these alterations were associated with an elevated risk of childhood asthma. An earlier study also linked lower fecal *Bacteroides* levels in infancy to the onset of atopy and wheezing by age five (Arrieta et al., 2018). Sphingosine can be converted into sphingosine-1-phosphate, which has been shown to regulate airway smooth muscle hyperreactivity and proliferation in asthma models (Maguire et al., 2023). In murine studi*es*, *Bifidobacterium*, as a probiotic, has been shown to modulate lung granulocytes by altering the metabolism of short-chain fatty acids such as tryptophan in the mice intestines (Bezemer et al., 2024), which concurrently alleviated allergic asthma symptoms (Wang et al., 2024). In addition, bile acid metabolites regulate host immune responses by modulating Th17/Tregs balance (Hang et al., 2019). In the study by Gürdeniz et al. (2022), cesarean-born offspring exhibited altered bile acid metabolism and elevated Treg levels. Furthermore, specific components of gut microbiota can modulate immune function, potentially disrupting Th17/Tregs and Th1/Th2 balance (Di Gangi et al., 2020; Irvin et al., 2014), ultimately predisposing individuals to allergic inflammation and asthma (Kotrba et al., 2024).

In terms of multiparous women and delivery methods, previous delivery history holds predictive value for the current delivery mode. Evidence indicates that women with a previous cesarean delivery are more likely to opt for repeat cesarean section. Conversely, multiparous women without a history of cesarean section generally prefer natural childbirth (Ayachi et al., 2016; ACOG, 2019). Moreover, siblings also play a significant role in influcing the development of the infant gut and respiratory tract microbiota. Their influence has been shown to surpass other early-life exposures including mode of delivery, breastfeeding, or antibiotic use. This early microbial enrichment leads to a distinctive “sibling-associated microbiome signature” by age one, which is associated with a reduced risk of asthma by age six (Christensen et al., 2022). Furthermore, birth order has been consistently found to be inversely correlated with allergic disease risk, indicating later-born children tend to have progressively lower risks of developing allergies (Bernsen et al., 2003; Luukkonen et al., 2024). Therefore, whether the mother is a multiparous woman, especially her previous delivery method, and sibling factors should be taken into full account in examing the impact of delivery methods on the infant gut microbiota or the risk of asthma.

Importantly, the impact of delivery mode on early-life microbiota appears to be time-sensitive (Rutayisire et al., 2016) and may also impose effects on subsequent feeding patterns (Prior et al., 2012). Previous studies have shown that cesarean delivery was associated with delayed initiation of breastfeeding, lower rates of exclusive breastfeeding, and an earlier transition to formula feeding (Prior et al., 2012; Alrasheedi, 2023). These changes in early feeding practices may alter the infant gut microbiome, as dietary exposures become the predominant determinants of microbial composition by around 13 weeks of age, with breastfeeding cessation associated with further compositional shifts (Galazzo et al., 2020). Taken together, these interaction between delivery mode, feeding patterns and their temporal dynamics suggest that there are potential critical windows during which delivery mode shapes microbial colonization. Future studies should prioritize longitudinal tracking of delivery mode–related microbial changes, particularly during the first three months of life and the weaning transition.

### Antibiotic

Epidemiological studies worldwide estimate that 20–30% of women are exposure to antibiotics during pregnancy (Loewen et al., 2018; Chandrakumar et al., 2018). The American College of Obstetricians and Gynecologists recommends routine prophylactic antibiotics before cesarean sections and selective use for vaginal births in high-risk cases (Committee on Practice Bulletins-Obstetrics, 2018). A study indicated limited impact of antibiotic administration during cesarean section on neonatal gut microbiome establishment (Sinha et al., 2024). These findings highlight the need for further investigation into the potential impacts of prenatal/pre-delivery antibiotic use on early microbial programming and the potential health outcomes.

According to Lee et al.’s study, prenatal antibiotic exposure was associated with small airway dysfunction and an increased asthma risk in offspring delivered by cesarean section (Lee et al., 2023). Animal models suggest that imbalanced maternal gut microbiota and reduced SCFAs concentrations may be key mechanisms linking prenatal antibiotic exposure to increased asthma severity in offspring (Alhasan et al., 2020; Alhasan et al., 2023). Butyrate deficiency downregulated type I interferon (IFN-I) signaling in neonatal type 2 innate lymphoid cells (ILC2s), increasing their responsiveness and promoting eosinophil infiltration and ILC2 numbers in the lungs. Changes in breast milk microbiota induced by antibiotics also influenced neonatal ILC2 phenotypes, contributing to offspring asthma development.

While prenatal antibiotics may affect offspring asthma via microbiota alterations, confounding factors especially delivery methods complicate epidemiologic interpretations. Future research should further explore whether antibiotic use at different stages of pregnancy exerts differential effects and emphasize rigorous adjustment for confoundere in study design.

### Maternal diet

As a key determinant of intestinal flora, maternal diet during pregnancy can influence the composition, diversity, and metabolic activity of the gut microflora (Barrientos et al., 2024). Dietary factors affecting gut microbiota can be categorized into four groups: dietary pattern, food components, vitamins and trace elements, and prebiotics. Changes in any group can profoundly impact gut microbiota and subsequently affect the immune system of both maternal and offspring generations (Hebert et al., 2021; Drall et al., 2020; Sindi et al., 2021). Existing review indicates that a high-fat diet during pregnancy may reduce gut microbial diversity, whereas higher fiber intake appears to be positively associated with it (Maher et al., 2023). In animal models, it has been further demonstrated that a low-fiber diet during pregnancy delayed plasmacytoid dendritic cell (pDC) recruitment and lung Tregs expansion, leading to an increase in lower respiratory tract infections in offspring (Rago et al., 2019). Lee-Sarwar et al. highlighted the pivotal role of SCFAs, particularly acetic acid (Lee-Sarwar et al., 2020), which are primarily produced by the fermentation of dietary fibers in the gut and serve as key mediators linking maternal diet to immune regulation and respiratory health outcomes in offspring (Deleu et al., 2021). In a mouse study, acetate supplementation reduced allergic airway inflammation, airway hyperresponsiveness, and inflammatory cell infiltration, likely by modulating immune responses via G protein-coupled receptors (Thorburn et al., 2015).

Given the long-term implications of maternal diet during pregnancy, future research should further explore differences in dietary patterns and food components, as well as whether the supplementation of vitamins, trace elements, and prebiotics will have more specific effects on the microbiota of both the mother and the offspring, and how microbiota differences affect offspring susceptibility to allergic diseases.

### Strengths and limitations

This review has several limitations to consider when interpreting the significance. First, despite a standardized search strategy, the potential omission of relevant studies cannot be excluded. Second, the included studies were categorized into four exposure groups, which could not be unified, limiting comparability. Third, although asthma was uniformly defined as the primary outcome, variations in follow-up duration led to inconsistencies in outcome evaluations. Moreover, substantial heterogeneity in microbial diversity measurements across studies complicated the identification of specific microbiome patterns associated with asthma development. Methodological variations also precluded both a formal quality assessment using standardized tools and a quantitative synthesis of results. In addition, most study populations were composed primarily of individuals of European descent. Only one study explicitly included race or ethnicity as an analytic covariate. These limitations underscore the importance of future studies employing standardized protocols for exposure assessment, outcome measurement, microbiome analysis among diverse populations.

## Conclusion

This review synthesizes emerging epidemiological evidence on the associations between prenatal exposures and offspring asthma, with a focus on the mediating role of the gut microbiota. By examining changes in infant gut microbial and immune development in response to prenatal factors, we clarify potential causal pathways. Specifically, prenatal pet exposure was associated with increased gut microbiota diversity in offspring, potentially decreasing the risk of asthma. In contrast, cesarean section was linked to reduced diversity of gut microbiota in infants, which could raise the risk of childhood asthma. Prenatal antibiotic exposure can disrupt gut microbiota balance in both mothers and offspring, thereby exacerbating asthma susceptibility. Maternal diet also played a role; high-fiber diets during pregnancy increased fecal short-chain fatty acids, especially acetate, which was shown to alleviate asthma in offspring.

Although heterogeneity in exposure definitions, microbial targets, and outcome assessments across studies limits the ability to draw unified conclusions, this review is the first to explicitly establish the link between prenatal exposures, offspring asthma, and the gut microbiome. Based on our findings, large-scale, multi-center longitudinal studies with extended follow-up are needed to systematically evaluate long-term asthma outcomes and early microbial alterations. In addition to environmental exposures, behavioral and psychological factors should be incorporated into exposure assessments. More cutting-edge methods are needed to identify microbial taxa, metabolites, and immune pathways linked to asthma development.
